# Autistic-like behavioral effects of prenatal stress in juvenile *Fmr1* mice: the relevance of sex differences and gene–environment interactions

**DOI:** 10.1038/s41598-022-11083-1

**Published:** 2022-05-04

**Authors:** Valeria Petroni, Enejda Subashi, Marika Premoli, Markus Wöhr, Wim E. Crusio, Valerie Lemaire, Susanna Pietropaolo

**Affiliations:** 1 INCIA, UMR5287, Bordeaux University and CNRS, 33000 Bordeaux, France; 2 Department of Molecular and Translational Medicine, University of Brescia, Brescia, Italy; 3 Research Unit Brain and Cognition, Laboratory of Biological Psychology, Social and Affective Neuroscience Research Group, Faculty of Psychology and Educational Sciences, KU Leuven, 3000 Leuven, Belgium; 4 Leuven Brain Institute, KU Leuven, 3000 Leuven, Belgium; 5 Behavioral Neuroscience, Experimental and Biological Psychology, Faculty of Psychology, Philipps-University of Marburg, 35032 Marburg, Germany; 6 Center for Mind, Brain and Behavior, Philipps-University of Marburg, 35032 Marburg, Germany; 7 INCIA, UMR 5287, Bordeaux University and CNRS, Bat B2, Allée Geoffroy St. Hilaire, CS 50023, 33615 Pessac Cedex, France

**Keywords:** Social behaviour, Stress and resilience, Autism spectrum disorders, Developmental disorders

## Abstract

Fragile X Syndrome (FXS) is the most common heritable form of mental retardation and monogenic cause of autism spectrum disorder (ASD). FXS is due to a mutation in the X-linked FMR1 gene and is characterized by motor, cognitive and social alterations, mostly overlapping with ASD behavioral phenotypes. The severity of these symptoms and their timing may be exacerbated and/or advanced by environmental adversity interacting with the genetic mutation. We therefore tested the effects of the prenatal exposure to unpredictable chronic stress on the behavioral phenotype of juveniles of both sexes in the Fmr1 knock-out (KO) mouse model of FXS. Mice underwent behavioral tests at 7–8 weeks of age, that is, when most of the relevant behavioral alterations are absent or mild in Fmr1-KOs. Stress induced the early appearance of deficits in spontaneous alternation in KO male mice, without exacerbating the behavioral phenotype of mutant females. In males stress also altered social interaction and communication, but mostly in WT mice, while in females it induced effects on locomotion and communication in mice of both genotypes. Our data therefore highlight the sex-dependent relevance of early environmental stressors to interact with genetic factors to influence the appearance of selected FXS- and ASD-like phenotypes.

## Introduction

Fragile X syndrome (FXS) is a neurodevelopmental disorder characterized by multiple behavioral alterations, including mental retardation, hyperactivity, anxiety, cognitive and social deficits^1^. Autistic symptoms, including altered social interaction and communication, are also often detected in FXS patients^2,3^: FXS is indeed considered as the most common monogenic cause of autism spectrum disorder (ASD). FXS is due to a mutation in the X-linked FMR1 human gene consisting in more than 200 CGG repetitions leading to the absence of FMRP protein^4^ playing a major role in synaptic and neuronal functionality^5^. The lack of FMRP has been recapitulated by the Fmr1-KO mouse model of FXS together with several relevant behavioral alterations^6^. The FXS-like behavioral phenotypes of mutant mice are mostly evident at adulthood, i.e., at 3–6 months, that is, when most preclinical studies are carried out (as reviewed in^7^).

Despite its clear and well-defined genetic origins, the FXS behavioral phenotype can be critically modulated by environmental factors, both in terms of its severity and of the timing of appearance. Environmental stimulation is for instance known to attenuate/delay the expression of behavioral alterations both in FXS patients and Fmr1-KO mice^8,9^. Conversely, exposure to stressful life events may exacerbate the behavioral deficits of FXS patients^10,11^, especially when occurring during early life phases. Exposure to prenatal stress is a powerful tool to induce early adversity in a genetic mouse model and therefore to study the impact of gene-environment interactions in the expression of its behavioral phenotype. Surprisingly, to our knowledge, the behavioral effects of prenatal stress have never been investigated in the Fmr1-KO mouse, or in other models of ASD (while they were demonstrated in genetic mouse models of neuropsychiatric and neurodegenerative disorders^12–14^).

Furthermore, prenatal stress is known to induce marked long-term behavioral alterations in wild-type rodents, including cognitive, emotional, motor and social abnormalities (reviewed in^15,16^). These studies have pointed out in particular the relevance of the unpredictable chronic mild stress procedure, as the most suitable experimental approach to model early environmental adversity in laboratory rodents^17–19^. This procedure, combining multiple stressors of different nature, has also the advantage to minimize habituation and exclude pain or nutritional effects^20,21^. In most existing preclinical studies (reviewed in^15,16^) stress exposure was implemented during the last week of gestation of the dams, as this phase is a preferential target to induce long-term brain and behavioral modifications in the offspring, because of its high environmental and stress sensitivity^22,23^.

The inclusion of mice of both sexes in the behavioral analysis of the offspring is considered of critical relevance for preclinical studies on prenatal stress exposure. Several sex differences have been indeed described in the behavioral response to stress in rodents; these include differences in the severity of stress effects, but also in their specificity to selected behavioral domains^13,24,25^. The inclusion of subjects of both sexes is also important for studying FXS, both in human and preclinical research. Although FXS is more common in boys than girls, increasing attention has been devoted to heterozygous females, as they are the ones producing the affected offspring^26^, and they represent the majority of FXS female patients, as homozygous *FMR1* mutations are extremely rare^27^. In humans, FXS female carriers present several behavioral symptoms, including hyperactivity^28^, mild cognitive impairments^29,30^ and autistic behaviors^31^. In mice, similar behavioral abnormalities were described in Fmr1 mutant females, especially at adulthood (as reviewed in^7^).

**Figure 1:**
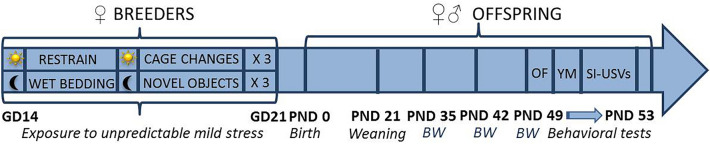
**Schematic representation of the experimental design of the study and its timeline.** Unpredictable mild stress consisted of the following 2 day-sequence that was repeated three consecutive times during the last week of gestation: on day 1, 3 sessions of 30-min restrain stress during the light phase, at 4 h intervals were followed by overnight housing with wet bedding, while on day 2, 3 sessions of sawdust and cage changes during the light phase, at 4 h-intervals, were followed by overnight housing with novel glass black beads. Control mice were left undisturbed during all pregnancy. Behavioral tests were conducted between 7 and 8 weeks of age, with 48hs interval between consecutive tests. GD = gestational day; PND = postnatal day; BW = body weight; OF = open field; YM = Y maze; SI = social interaction; USVs = ultrasonic vocalizations.

Here we therefore evaluated whether exposure to unpredictable chronic mild stress during the last prenatal week could advance and/or exacerbate the juvenile behavioral phenotype of Fmr1-KO offspring of both sexes (as schematized in Fig. 1). To this end, Fmr1-KO male (hemizygous, -/Y) and female (heterozygous, +/−) mice, together with their WT littermates, underwent behavioral tests for exploration, spatial memory, social interaction and communication at the juvenile age of 7–8 weeks, i.e., when most of the FXS-like behavioral alterations are absent or mild. At this age, Fmr1-KO males do not show any remarkable behavioral phenotype in the considered domains^7,32^, while mutant females displayed mild alterations in social interaction and communication^33^. This age partially overlaps with adolescence (occurring between 3 and 8 weeks of age in mice), a critical phase for brain and behavioral development in rodents and humans and largely involved in several neuropsychiatric disorders^34^. This phase has been also extensively studied for the expression of social behaviors in laboratory mice, with a special emphasis on the post-pubertal phase (i.e., approximately after the 5 weeks of age), since it is characterized by important changes in the patterns of intra-specific social interactions^35^. Late adolescence (7–9 weeks) is also of particular interest, since most behavioral abilities are already well developed in mice; it is therefore suitable to multiple behavioral testing, performing the same cognitive, emotional, and social tests done in adult mice and hence facilitating comparisons with data from adult subjects.

## Methods

### Ethics approval

All experimental procedures were in accordance with ARRIVE guidelines (https://arriveguidelines.org), European Communities Council Directive 2010/63/EEC. Furthermore, there were approved by local ethical committee (“Comité d’Ethique pour l’experimentation animale de Bordeaux”, CE 50) and the French Ministry (“Ministere de l’enseignement superieur de la recherché et de l’innovation”).

### Breeding and stress procedure

Twenty adult (12 ± 1 weeks-old) virgin Fmr1 heterozygous (+/−) females and 10 C57BL/6J adult wild type males [16 weeks-old; purchased from Janvier (Le Genest St Isle, France)] were used as breeders to generate the tested offspring. C57BL/6JFmr1^tm1Cgr/Nwu^ (B6) mice were originally obtained from Neuromice.org (Northwestern University) and maintained on the C57BL/6/J background for more than 10 generations.

They were bred as described previously^32^. Each half of the female breeders was assigned to one of the following groups in which they were kept during the last week of pregnancy: no-stress, i.e., kept undisturbed in their home-cage, or stress, i.e., exposed to the unpredictable stress procedure described below.

The time line of the study is illustrated in Fig. 1. The stress procedure included the following 2-day sequence of events that was repeated three consecutive times during the last week of gestation:

- Day 1: 30 min of restrain stress (3 times each day during the light phase, with a 4 h-interval) in perforated conical tubes (3 cm in diameter, 11.5 cm long; Becton Dickinson Labware Europe, France), followed by overnight housing with wet bedding (50 ml of water were added to floor sawdust of the home cage at the beginning of the dark phase).
- Day 2: multiple sawdust and cage changes (3 times each day during the light phase, with a 4 h-interval), followed by overnight housing with novel objects (12 glass black beads, 1.5 cm in diameter were added in the home cage at the beginning of the dark phase).

Pregnant females were exposed to this sequence of events for 3 times during the last week before parturition: this procedure is based on previous studies (e.g.^23,36–38^) and it is known to limit the habituation to stressful stimuli without using pain or nutritional manipulations. All breeders used for the study gave birth within 48hs after the last day of exposure to stress procedure. They were left undisturbed until weaning of the pups, i.e., on post-natal day (PND) 21. No alteration in the general health status of stressed breeders emerged at the end of the stress paradigm. The health measures were taken by the animal caretakers through the daily observation of the animals in their home cage in order to assess both behavioral and physical indicators of welfare^39^. These included hunched posture, dull or sluggish movements, reduced locomotion/immobility, altered nest building and stereotypic behaviors, excessive grooming, absence of feces, rough hair coat, squinted eyes, skin abrasions/lesions^39^.

### Animals and housing procedures

At 3 weeks of age, all pups were weaned and housed in same-sex groups of 3–5 littermates in our animal facility^9,32^. On the same day, tail samples were collected for DNA extraction and subsequent PCR assessment of the genotypes as previously described^6^. Mice were then left undisturbed until the beginning of behavioral testing (i.e., at 7 weeks of age), except for the evaluation of body weight that was carried out once a week starting at 5 weeks of age (Fig. 1). Only litters including males and females of both mutant (KO for males and HET for females) and wild-type (WT) genotypes were used for experiments, for a total of 14 litters. A total of 93 mice were subjected to behavioral testing: 45 males [25 WT and 20 KO (-/Y), n = 9–15 for stress condition) and 48 females [24 WT and 24 HET (+/−), n = 12 for stress condition].

Stimulus mice used for the direct social interaction test were adult (12 weeks of age) female NMRI mice, as this strain is commonly employed in social studies^40,41^, especially those using the Fmr1-KO mouse model^9,32,33^. This strain is often chosen since it is characterized by high levels of sociability, and it facilitates the behavioral analysis during social encounters with B6 mutants because of its albino phenotype. NMRI mice were purchased at 10 weeks of age from Janvier (Le Genest-Saint-Isle, France), housed in groups of 3–4 per cage and left undisturbed for 2 weeks before being used in behavioral tests. The choice of the age of stimulus mice was based on previous studies with male mice (both adults and juveniles; e.g.^42–47^), and with females in the resident-intruder setting^41,48,49^, all using adult stimulus females. Indeed, in these experimental contexts, adult stimulus females do not emit ultrasonic vocalizations (USVs) that are instead mostly uttered by the experimental male^44,48^ or resident female^48,49^, as demonstrated by alternately anesthetizing each pair member. For these reasons, we have previously used an adult female stimulus to assess ultrasonic communication in juvenile Fmr1 mice of both sexes^32,33^. Indeed, juvenile females are known to produce a high number of USVs during same-age interactions^50^, both with male and female experimental mice. During juvenile-juvenile interactions in mice, both pair members are indeed supposed to emit USVs, so that the USVs of each pair (usually chosen with matching characteristics) represent the only variable taken into consideration (e.g.,^50^). This situation is easily detectable by spectrographic analysis, through the identification of “double calls”, i.e., overlapping in their timing, but with different, non-harmonic, characteristics (e.g., different peak and mean frequency, modulation). Here the presence of these double calls was excluded by the additional inspection of all spectrograms.

### Behavioral testing procedures

Behavioral tests commenced at 7 weeks of age and were conducted as follows (see also Fig. 1). On day 1, an open field test for locomotion and exploration was administered, followed on day 3 by a spontaneous alternation test in a Y-maze, and on day 5 by a direct social interaction test and the females’ estrous cycle assessment. All behavioral tests were carried out during the light phase of the cycle (between 9 a.m. and 4 p.m.) by an experimenter who was blind to the group assignment of the subjects. All mice were habituated to the experimental room for at least 30 min before the beginning of each behavioral test.

#### Open field

The open field^32^ consisted of a white plastic arena (42 × 26 × 15 cm) where the locomotion of each mouse was assessed during 10 min using automated tracking (Ethovision, Noldus, The Netherlands).

#### Y maze

The Y maze test (described in details before^32^) was employed to assess spontaneous alternation through a 2-trial procedure, consisting of a 5-min habituation trial, followed by a 2-min test trial. Time spent in each arm during the habituation and testing phases was scored by automatic tracking and percent alternation rates during the test phase were derived as follows: 100 × (time in novel arm/time in all arms).

#### Social interaction and ultrasonic communication

Male experimental subjects were habituated to the testing apparatus^9,32^ for 30 min prior to testing, while female subjects were isolated in the testing cage for 72hs, in order to induce a status of resident in experimental females and therefore promote the emission of ultrasonic vocalizations (USVs) towards an adult female intruder^41^. An unfamiliar stimulus female mouse (an adult NMRI female) was then introduced into the testing cage of either male or female subjects and left there for 3 min.

Testing sessions were recorded by a camera placed on the side of the cage and videos analyzed with Observer XT (Noldus, The Netherlands). One observer who was unaware of the genotype and sex of the animals scored the behavior of the test mice, quantifying the time spent performing affiliative behaviors^9,32,33^, i.e., sniffing the head and the snout of the partner, its anogenital region, or any other part of the body; contact with the partner through traversing the partner’s body by crawling over/under from one side to the other or allogrooming. Nonsocial activities were also measured^9,32^: rearing (standing on the hind limbs sometimes with the forelimbs against the walls of the cage); digging; self-grooming (the animal licks and mouths its own fur). An ultrasonic microphone UltraSoundGate Condenser Microphone CM 16 (Avisoft Bioacoustics, Berlin, Germany) was mounted 2 cm above the cover of the testing cage. Recordings were then analyzed through Avisoft SASLab Pro (Version 5.20; Avisoft, Berlin, Germany) to compute the number of USVs as well as their mean duration, peak frequency and peak amplitude^9,32^. In addition density plots depicting the distribution of total calls for each genotype at peak frequency versus duration were obtained as described in details elsewhere^51,52^. Call subtypes were also determined for a more detailed qualitative analysis; for this purpose, USVs were automatically classified using the Sonotrack Call Classification Software (version 1.4.7, Metris B.V., The Netherlands), using categories previously described in details elsewhere^46^.

The estrus phase of female mice was assessed by analysis of vaginal smears^53^ performed on the testing day in both the experimental subjects and NMRI stimulus mice. The evaluation of Fmr1 WT and HET (+/−) females used as experimental subjects was conducted after their testing, in order to minimize the potential stress effects of the manipulation necessary for determining the estrous phase. Stimulus NMRI females were approximately half in diestrus and half in estrus phases, and their assignment to social encounters was equally distributed between experimental groups. The estrus phase of experimental female subjects included pro-estrus, estrus and diestrus, following a distribution that was balanced across genotypes and stress conditions.

### Statistical analysis

All data were separately analyzed in males and females. This was due to sex differences in (i) the X-linked Fmr1-mutation (i.e., hemizygous in males, heterozygous in females), (ii) in some behavioral testing procedures (such as different duration of pre-testing isolation necessary for USV assessment), (iii) in most of the behavioral phenotypes measured here. The latter sex differences were further confirmed in our data set, through a preliminary ANOVA showing overall sex effects in basically all measured variables (data not shown).

Data from each sex were analyzed with a 2 × 2 ANOVA with genotype and stress as the between subject factors. Within-subject factors were included when appropriate (e.g., testing time for body weight). Alternation rates from the Y-maze test were instead analyzed for differences from the chance level (with a t-test), in line with previous studies^54^. Post-hoc comparisons were performed using Fisher’s LSD test when a significant interaction was detected. Separate ANOVAs were also conducted when appropriate. Data from the density plots of ultrasonic calls did not undergo statistical analysis, but were used to obtain a qualitative three-dimensional evaluation of USV data^51,52^.

Analyses were conducted using the software Statview and SPSS and α was set at 0.05. Results are expressed as mean ± SEM throughout the text. The exact number of mice is indicated in the legend of each figure; differences may be due to technical reasons (e.g., loss of behavioral video recordings) or to the exclusion of outliers (using Grubbs' ESD test adapted for small sample size) or of non-vocalizing mice for USV assessment (these included a total of 4 males and one female).

## Results

### Body weight

**Figure 2:**
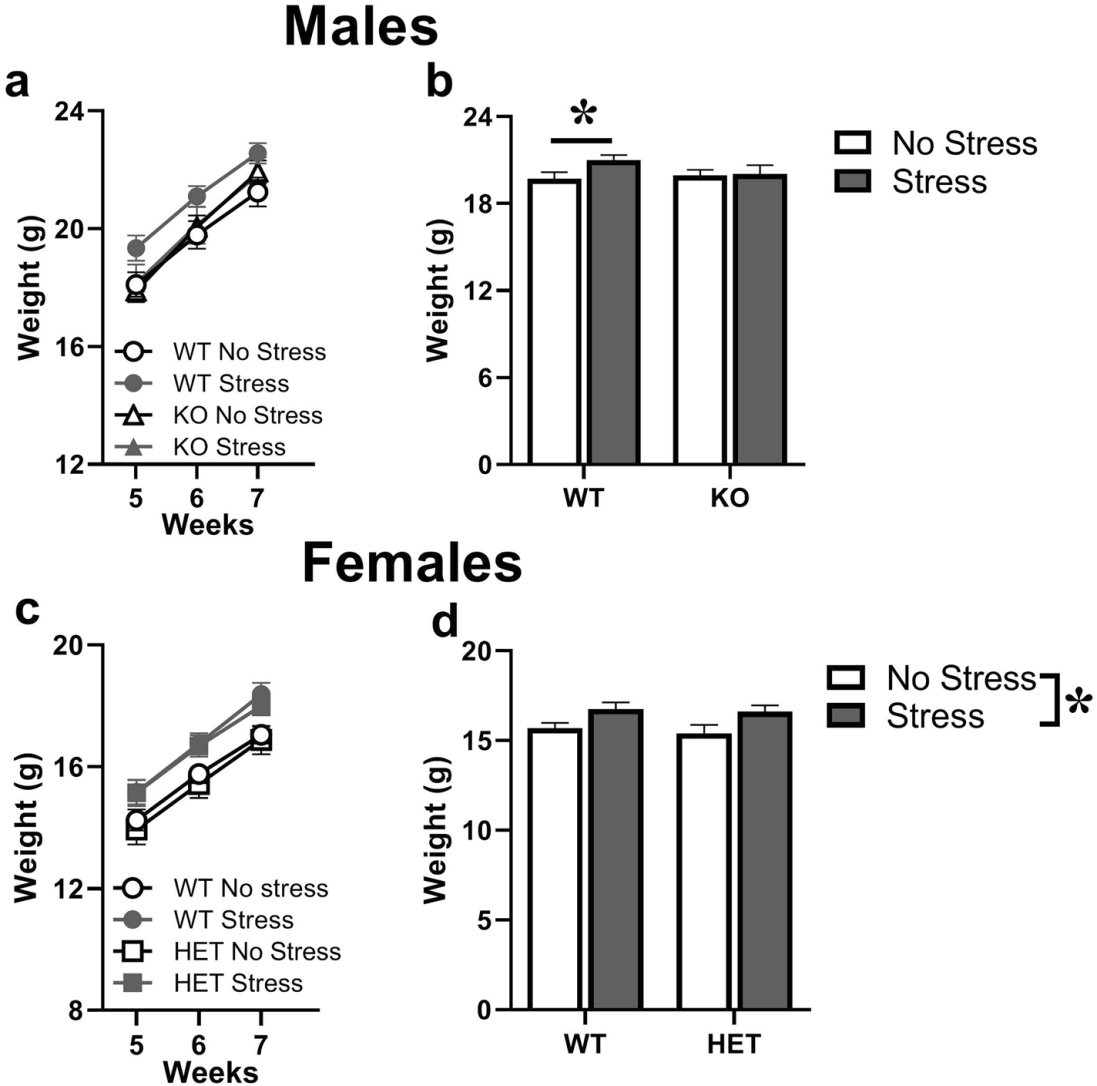
**Effects of prenatal stress in juvenile mice on body weight.** Body weight was assessed during the last two weeks before behavioral testing, i.e., at 7 weeks of age. Time course illustrates the expected weight gain in males and females (**a**–**c**), while overall group differences are shown by the mean weight values averaged across time-points in each sex (**b**–**d**). *p < 0.05. N for males: 15 WT-no stress, 10 WT-stress, 9 KO-no stress, 11 KO-stress; N for females: 12 in all groups. KO refers to -/Y in males, HET to +/− in females. Data are expressed as mean ± SEM.

Body weight was assessed once a week between 5 and 7 weeks of age (Fig. 2). In males, there was an expected body weight gain with time [testing time effect: F(2,82) = 982.57, p < 0.0001; Fig. 2a] and this was more marked in WT mice than KOs [interaction genotype × time: F(2,82) = 9.22, P < 0.001]. Nonetheless, this was mainly due to the overall higher body weight of WT-stressed males, as demonstrated by separate ANOVAs showing a significant effect of stress in WT mice only [F(1,23) = 4.2, p = 0.05; in KO: n.s.; Fig. 2b]. A similar pattern was found in females, where body weight also increased over weeks as expected [time effect: F(2,88) = 768.32, p < 0.0001; Fig. 2c], and this gain did not differ between genotype or stress conditions [all interactions with time, ns]. In females also, stress increased the overall body weight, but equally in both WT and HET mice [main stress effect: F(1,44) = 9.17, p < 0.01; Fig. 2d].

### Open field

**Figure 3:**
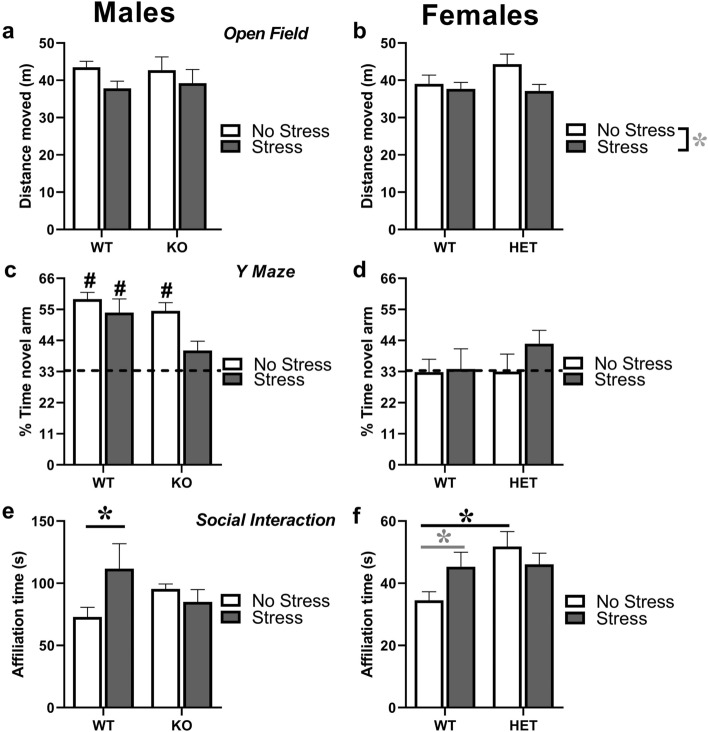
**Behavioral effects of prenatal stress in juvenile mice.** Locomotion was assessed in the open field test (**a**,**b**), while spontaneous alternation was evaluated in the Y maze (**c**,**d**). Social interaction was measured during a 3-min encounter with an adult NMRI WT female (**e**,**f**). *p < 0.05; * in grey p = 0.06; ^#^p < 0.05 versus chance level (indicated by dotted line). N for males: 14 (**a**,**e**) or 13 (**c**) WT-no stress, 9 (**a**,**c**,**e**) WT-stress, 8 (**a**,**e**) or 9 (**c**) KO-no stress, 11 (**a**,**e**) or 10 (**c**) KO-stress; N for females: 12 in all groups (**b**,**d**,**f**). KO refers to -/Y in males, HET to +/− in females. Data are expressed as mean ± SEM.

In males, there was no difference among experimental groups in locomotor activity in the open field [genotype, stress effects and their interaction: all n.s.; Fig. 3a]. In females, a tendency to a decrease in locomotor activity following stress was observed in mice of both genotypes [stress effect: F(1,44) = 3.87, p = 0.060; Fig. 3b].

### Y-maze

All male and female mice equally explored the maze arms during the habituation phase, and no differences among experimental groups were detected (data not shown). During the test phase, all males displayed spontaneous alternation, except stressed KO mice that showed a performance not significantly different from the chance level: (t = 2.16, ns; in other groups, all ts > 4, p < 0.01; Fig. 3c). In females, none of the four experimental groups showed significant levels of spontaneous alternation (t-tests: all ns; Fig. 3d), suggesting that this cognitive ability is not sufficiently expressed in *Fmr1* WT and HET female mice at this juvenile age.

### Social interaction

In males, WT stressed mice showed higher levels of affiliative behaviors towards the WT female stimulus [interaction genotype × stress: F(1,38) = 4.47, p < 0.05; post-hoc: WT-no stress versus WT-stressed, p < 0.05; Fig. 3e]. In females, HET mice showed enhanced levels of affiliation towards the WT female intruder compared to their WT littermates, but this genotype difference disappeared following stress, since stress tended to increase affiliative levels in WT mice [interaction genotype × stress: F(1,44) = 4.19, p < 0.05; post-hoc: WT-no stress versus HET-no stress, p < 0.05; WT-no stress versus WT-stressed, p = 0.06; Fig. 3f]. No significant effects were found for any non-social behaviors in both sexes (data not shown).

### Ultrasonic vocalizations (USVs)

**Figure 4:**
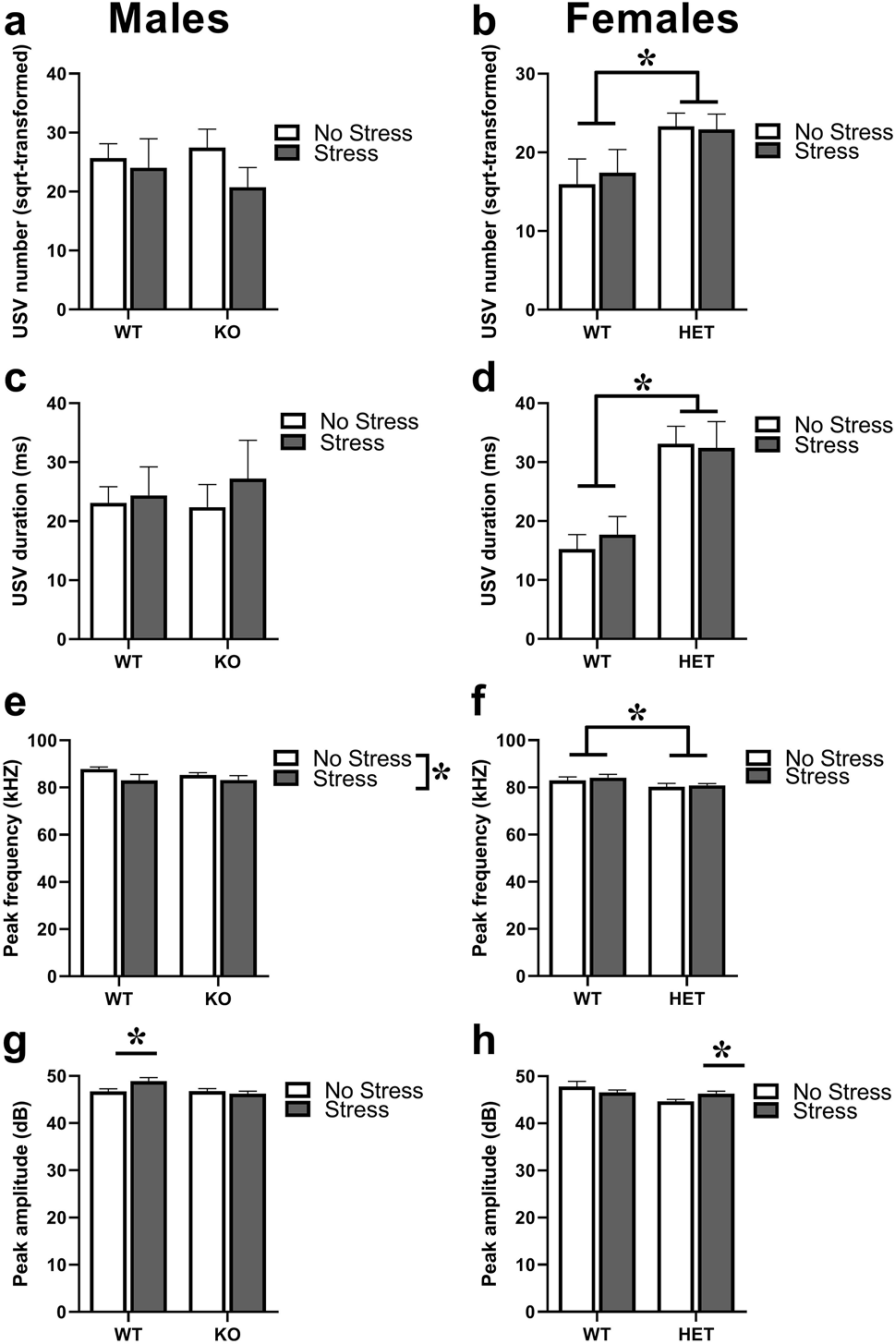
**Effects of prenatal stress on ultrasonic communication in juvenile mice.** Ultrasonic vocalizations (USVs) were assessed during the direct social interaction test with an adult NMRI WT female in Fmr1 mice of both sexes. The following parameters were measured through spectrographic analysis of the calls: total number (**a**,**b**), mean duration (**c**,**d**), mean peak frequency (**e**,**f**) and amplitude (**g**,**h**). The number of the calls was subjected to square-root (sqrt) transformation in order to meet the normality assumptions of parametric ANOVA. * p < 0.05. N for males: 10 (**a**,**c**,**e**,**g**) WT-no stress, 8 (**a**,**c**,**e**) and 7 (**g**) WT-stress, 8 (**a**,**c**,**e**) and 11 (**g**) KO-no stress, 10 (**a**,**c**,**e**,**g**) KO-stress; N for females: 11 (**b**,**d**,**f**,**h**) WT-no stress, 12 (**b**,**d**,**f**,**h**) WT-stress, 12 (**b**,**d**,**f**) and 11 (**h**) KO-no stress, 12 (**b**,**d**,**f**) and 11 (**h**) KO-stress. KO refers to -/Y in males, HET to +/− in females. Data are expressed as mean ± SEM.

In males, the number of USVs and their mean duration did not differ among experimental groups [genotype, stress effects and their interaction: all ns; Fig. 4a,c]. Stress decreased the mean peak frequency in mice of both genotypes [F(1,32) = 4.50, p < 0.05; Fig. 4e] and contribute to the emergence of a significant genotype difference in the mean peak amplitude, due to the highest values of WT-stressed mice [interaction genotype × stress: F(1,30) = 5.22, p < 0.05; post-hoc: WT-no stress versus WT-stressed, p < 0.05; Fig. 4g]. In females, HET mice emitted more and longer USVs compared to WT animals, and this effect was not altered by stress exposure [genotype effect on number (sqrt-transformed) and mean duration, respectively: F(1,43) = 6.65, 23.42, p < 0.05 and 0.0001 (Fig. 4b,d); all other effect and interactions: ns]. USVs produced by HET females were also characterized by a significant lower mean peak frequency [genotype effect: F(1,43) = 5.38, p < 0.05; Fig. 4f] and by lower peak amplitude, but only under no stress conditions [genotype × stress interaction: F(1,41) = 4.81, p < 0.05; post-hoc WT-no stress versus HET-no stress, p < 0.05; Fig. 4h].

**Figure 5:**
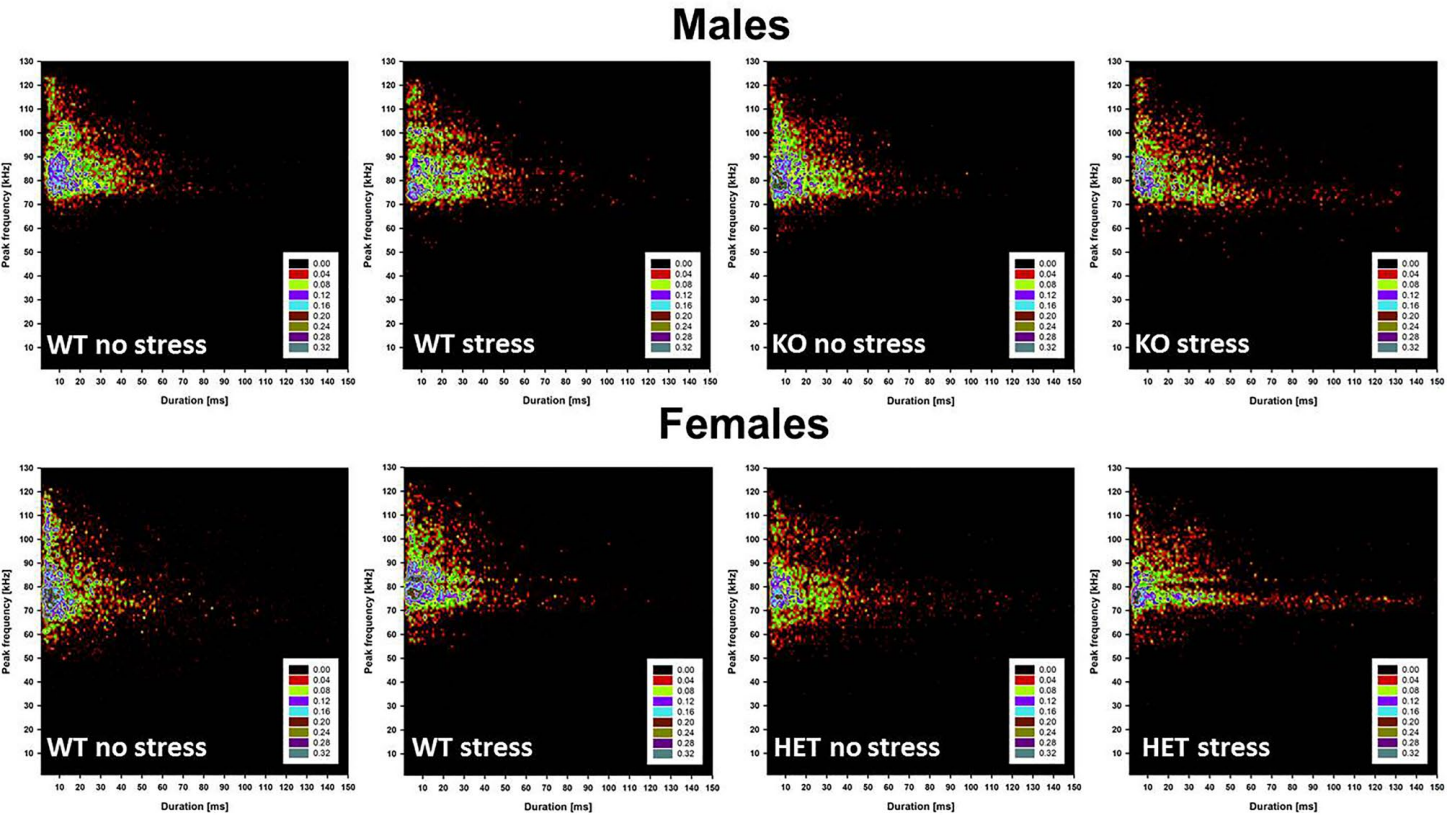
**Density plots of individual ultrasonic calls.** Density plots depict the distribution of individual USV emitted during 3-min social interaction with a NMRI adult stimulus female, plotted by frequency in kHz and duration in ms. Color coding reflects frequency in percentages.

The inspection of the density plots (Fig. 5) extended the results previously obtained from the quantitative analyses of the ultrasonic spectrograms. In both males and females, stress tended to increase the occurrence of unusual long USVs (mean duration > 60 ms, Fig. 5) an effect that appeared especially marked in mutant mice. In KO/HET-stressed mice there was an increased variability in the duration of the calls, an effect that was particularly dramatic in females (Fig. 5 lower panel).

**Figure 6:**
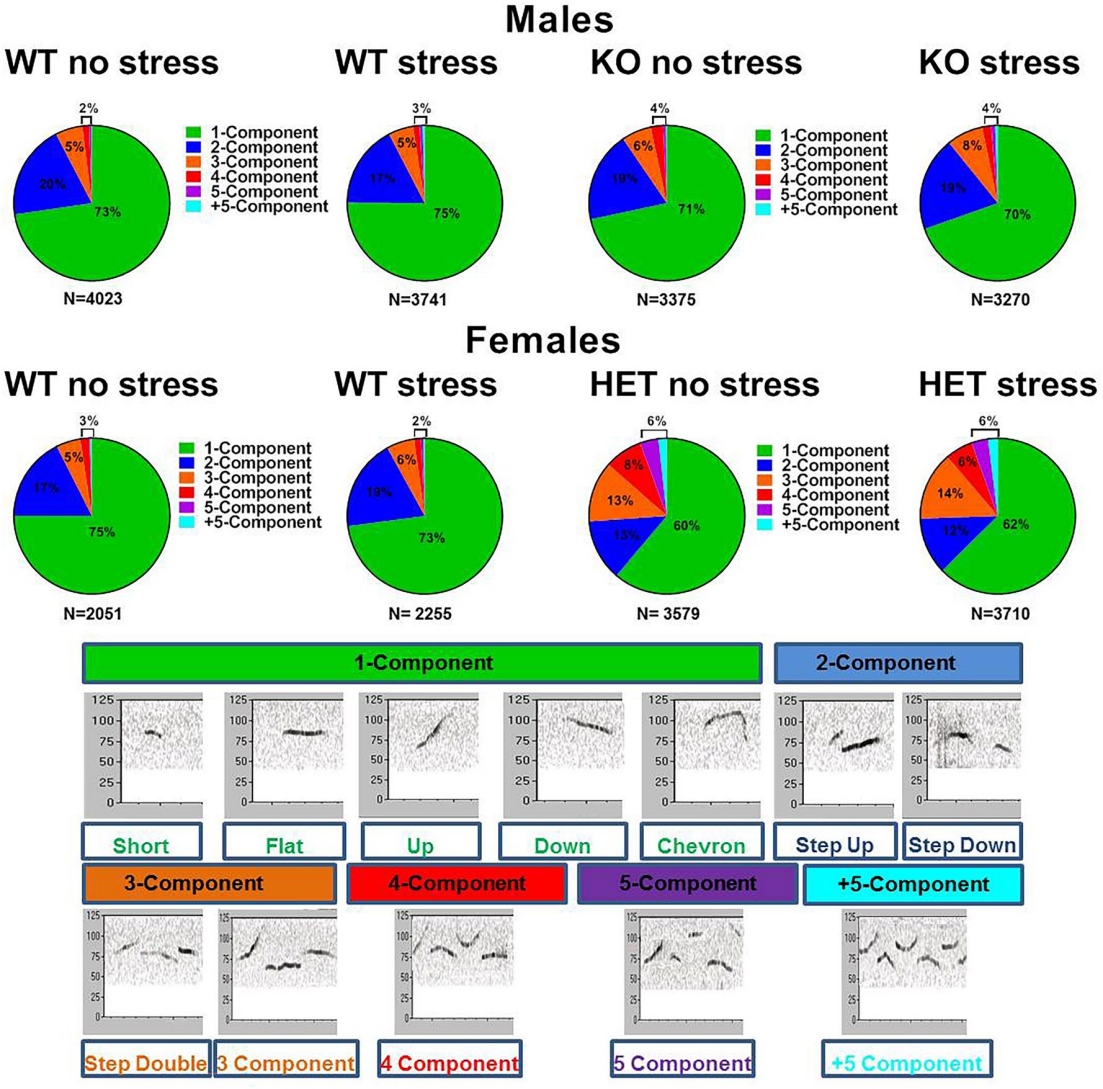
**Composition of ultrasonic call types.** Pie charts illustrate the different call types automatically classified by Sonotrack software. Call categories are expressed as percentages over the total number of USVs (N) for each experimental group.

The analysis of call subtypes^46^ revealed no major difference in the composition of the calls emitted by males [genotype, stress effects and their interaction, all n.s.; Fig. 6). In contrast, a clear genotype difference emerged in female mice, irrespectively of their stress conditions (Fig. 6). Female Fmr1-HETs emitted less simple calls, i.e., based on one or two components [genotype effects, respectively: F(1,42) = 18.06 and 14.59, p < 0.001], and more complex calls, i.e., containing 3, 4, 5 or more components, than their WT littermates [genotype effects, respectively: F(1,42) = 57.21, 58.48, 35.16 and 26.26, p < 0.0001]. This is in line with the results of the density plots, since complex calls typically correspond to longer USVs.

## Discussion

Our findings highlighted the impact of several gene-environment interactions on the behavioral phenotype of juvenile Fmr1 mutant mice that varies according to the sex of the animals, as summarized by Table 1. Overall, prenatal exposure to stress was able to induce several effects that were mostly dependent on sex differences and the considered behavioral domain. Our hypothesis, i.e., that stress exposure may advance/exacerbate the emergence of the behavioral alterations of Fmr1-KO mice was only partially confirmed, i.e., in the cognitive domain of spontaneous alternation and in male mice (Table 1).

**Table 1:** **Summary of the results.**

Variables measured	♂		♀	
	KO genotype effect	Stress effect	HET genotype effect	Stress effect
Body weight (Fig. 2)	–	**↑only in WT**	–	**↑** in WT and HET
Locomotion (Fig. 3a,b)	–	–	–	**↓**in WT and HET
Spontaneous alternation (Fig. 3c,d)	–	***↓only in KO***	–	–
Social interaction (Fig. 3e,f)	–	**↑only in WT**	**↑ in no stress**	**↑ only in WT**
Ultrasonic communication (Figs. 4, 5 and 6)	–	↓peak frequency in WT and KO **↑ peak amplitude only in WT**	**↑**call number and duration, ↓peak frequency, ↑complex calls, ↓ simple calls	–
				–
				**↑ peak amplitude only in HET**

All gene-environment interactions are marked in bold; italics bold refers to interactions inducing the emergence of a novel KO/HET phenotype (i.e., different from WT) under stressed conditions.

As expected from previous reports^7^, our results confirmed that the behavioral phenotype of our juvenile Fmr1-KO mutants was almost undistinguishable from their WT littermates. This is the reason why we chose this testing age as it provided the optimal baseline conditions to evaluate a potential exacerbating/anticipating impact of prenatal stress avoiding floor or ceiling effects. In male KO mice, no alteration emerged in any of the considered behavioral domains under no stress conditions, supporting the view that FXS- and ASD-like behavioral abnormalities, such as hyperactivity, cognitive deficits and social alterations, appear only at adulthood^7^. In females, an hyper-social phenotype was the only one detected in our juvenile mutants, including enhanced affiliation levels, increased number of ultrasonic calls and their duration (with qualitative alterations). Once again, these results were in agreement with our previous reports: interestingly, these communicative and social abnormalities were observed only at the juvenile age as they disappeared in adult mutant females^33^. These hyper-social phenotypes may seem surprising in view of the ASD-like alterations shown by FXS patients, consisting mostly of social avoidance and reduced social interest. Nonetheless, the more abundant and longer USVs emitted by mutant juvenile females could be also interpreted as autistic-like phenotypes, since several studies have described excessive talking and repetitive speech as major autistic communicative alterations in FXS patients (see for example^28^). Furthermore, our analysis of the ultrasonic call types revealed a different composition of the USV repertoire of Fmr1-HET females (Fig. 6), with a prevalence of complex multi-component calls compared to WT littermates. Although little is still known about the social meaning of different call types^46^, it is possible that Fmr1-HET females may emit more and longer USVs, but with less appropriate or adaptive communicative properties. The increased levels of affiliations could also be interpreted as an inappropriate social attitude since they are directed toward an intruder, i.e., a potential threat for the resident female. This testing context was indeed necessary to allow the detection of USVs in female mice^41^. It is therefore still possible that a different social phenotype may appear in a different testing context, e.g., in a neutral environment; indeed, when Fmr1 mutant juvenile females were assessed for their social interest in the three compartment test no sign of increased sociability was observed^33^.

On this basis of genotype differences, prenatal exposure to stress was able to induce the appearance of a cognitive deficit in the spontaneous alternation Y maze test, although only in males. KO stressed male mice were indeed the only experimental group displaying a performance similar to the chance level (Fig. 3c; Table 1). In females, stress instead seemed to eliminate the hyper-social phenotype of mutant mice (Fig. 3e) without affecting their ultrasonic communication profile (Fig. 4). Nonetheless, these effects in females were actually due to a selective effect of stress in WT mice, rendering the WT phenotype similar to that of mutants. Hence, our data suggest that exposure to prenatal stress does not dramatically advance the appearance of pathological behavioral phenotypes in male and female mutants, juvenile stressed KO/HET mice being mostly comparable to their WT littermates, as in no-stress conditions. Indeed, with the exception of the Y maze effect in males, no selective effect of stress on mutant behavioral phenotypes was detected (Table 1). Our findings may therefore suggest a higher sensitivity of the cognitive domain to the effects of stress in the male sex, in line with clinical data describing a positive correlation between stress levels and cognitive deficits in FXS boys^55^. Nonetheless, additional memory tests other than the Y maze for spontaneous alteration would be useful to fully confirm the selective efficacy of prenatal stress in the cognitive domain, an issue that could be specifically addressed in future studies combining spatial and non-spatial memory tests.

Interestingly, stress did interact with genotype on several behavioral measures, but mostly by inducing its effects in WT mice only. This may suggest a reduced sensitivity of Fmr1-KO/HET mice to stress that could be interpreted as a deficit in the adaptive response to stressors, as already proposed by others^56^. Previous studies have indeed described a reduced behavioral and endocrine sensitivity of adult Fmr1-KO mice (though only males were investigated) to the post-natal exposure to chronic stressors^56,57^. Here the genotype-specific effects of stress were characterized by clear sex differences: in males, stress enhanced body weight (Fig. 2a), affiliative behaviors (Fig. 3e) and peak amplitude (Fig. 4g) in WT only, while it reduced peak frequency in both genotypes (Fig. 4e). In females, stress enhanced affiliative behaviors in WT only (Fig. 3f), while it enhanced body weight (Fig. 2b) and reduced locomotion in both WT and mutant mice (Fig. 3b). Furthermore, in female HETs stress increased USV peak frequency (Fig. 4f). Overall, not the magnitude, but the behavioral specificity of the effects of stress differed between sexes, in line with most of the previous reports^58–62^.

The promoting effects of stress on social interaction were observed in WT mice of both sexes and may be explained by multiple hypotheses. One possible explanation lies in the prosocial effects of increased oxytocin, since this has been described in hypothalamic and limbic brain regions following exposure to a variety of stressors^63^. A second possible interpretation may consider the increased social interaction of WT stressed mice as a reflection of a the altered excitatory/inhibitory(E/I) imbalance induced by stress especially in brain circuits involving the prefrontal cortex^15,64^, known to critically control social behaviors in rodents^65,66^. Our findings suggest that these potential changes in oxytonergic or E/I systems are in any case induced by stress only in WT mice, perhaps because of a reduced functionality of these adaptive mechanisms of stress response in our Fmr1 mutant animals.

Despite the overall agreement of the behavioral effects described in WT mice by our findings, an important difference between our and others’ studies on prenatal stress should be underlined, that is, the genotype of our breeders exposed to prenatal stress. The dams exposed in our study to prenatal stress are indeed heterozygous Fmr1 mutant females and not WT as in previous similar studies: it is therefore possible that the sensitivity to stress of our female breeders may be different (as previously demonstrated for Fmr1-KO males with adult post-natal stress^56,57^) and result in specific sex-dependent effects on the offspring behaviors. Studies comparing the behavioral and endocrine response to stress of Fmr1 mutant and WT dams should be performed in the future in order to clarify this issue; also, it would be interesting to evaluate the maternal behavior of stressed and no-stress dams to investigate whether the effects of stress on the Fmr1 offspring behavior could be mediated by alterations in the maternal care received. Similarly to other manipulations of the early environment (e.g., early enrichment^67^), prenatal stress may induce its effects on the offspring both at the prenatal level, i.e., directly affecting pups’ embryonic development, and during the early post-natal phase, i.e., interfering with normal mother–pup interactions and altering maternal behaviors^68^.

In conclusion, our findings demonstrate for the first time the impact of prenatal stress on the juvenile FXS- and ASD-like behavioral phenotype of Fmr1 mice, underlying the relevance of including sex differences and assessing multiple behavioral domains in mouse studies on FXS and ASD. These data therefore highlight the importance of complex gene-environment interactions in the etiopathology of neurodevelopmental disorders, also for a syndrome of clear genetic origins, such as FXS. The early timing of the stress exposure used here may be of critical relevance, since previous studies using post-natal chronic stress paradigms in the same mouse model showed less varied and marked effects on FXS-like neurobehavioral phenotypes^56,57^. Our results also focused on the juvenile age, which is critical for the early detection of behavioral abnormalities and their early therapeutic rescuing; this research focus could be extended in future studies by investigating the effects of prenatal stress on a longer term, for instance on the behavioral phenotype of Fmr1 mice at the adult age, i.e., when the behavioral alterations of mutants are more marked and well-established.

## Data Availability

The datasets used and analysed during the current study are available from the corresponding author on reasonable request.

## References

[1] Hagerman RJ, Hagerman PJ (2002). Fragile X Syndrome: Diagnosis, Treatment, and Research.

[2] Bailey DB (1998). Autistic behavior in young boys with fragile X syndrome. J. Autism Dev. Disord..

[3] Hagerman RJ (2006). Lessons from fragile X regarding neurobiology, autism, and neurodegeneration. J. Dev. Behav. Pediatr..

[4] Pieretti M (1991). Absence of expression of the FMR-1 gene in fragile X syndrome. Cell.

[5] Greenough WT (2001). Synaptic regulation of protein synthesis and the fragile X protein. Proc. Natl. Acad. Sci. USA.

[6] Dutch-Belgian Fragile X Consortium (1994). Fmr1 knockout mice: A model to study fragile X mental retardation. Cell.

[7] Pietropaolo, S. & Subashi, E. In *Behavioral Genetics of the Mouse* Vol. 2 (eds Pietropaolo, S. *et al.*) 146–163 (Cambridge University Press, 2014).

[8] Dawson G (2002). Defining the broader phenotype of autism: Genetic, brain, and behavioral perspectives. Dev. Psychopathol..

[9] Oddi D (2015). Early social enrichment rescues adult behavioral and brain abnormalities in a mouse model of fragile X syndrome. Neuropsychopharmacology.

[10] Dyer-Friedman J (2002). Genetic and environmental influences on the cognitive outcomes of children with fragile X syndrome. J. Am. Acad. Child. Adolesc. Psychiatry.

[11] Hessl D (2001). The influence of environmental and genetic factors on behavior problems and autistic symptoms in boys and girls with fragile X syndrome. Pediatrics.

[12] van den Hove DL (2011). Differential effects of prenatal stress in 5-Htt deficient mice: Towards molecular mechanisms of gene x environment interactions. PLoS ONE.

[13] Sierksma AS (2013). Behavioral and neurobiological effects of prenatal stress exposure in male and female APPswe/PS1dE9 mice. Neurobiol. Aging.

[14] Oliver PL, Davies KE (2009). Interaction between environmental and genetic factors modulates schizophrenic endophenotypes in the Snap-25 mouse mutant blind-drunk. Hum. Mol. Genet..

[15] Sandi C, Haller J (2015). Stress and the social brain: Behavioural effects and neurobiological mechanisms. Nat. Rev. Neurosci..

[16] Weinstock M (2008). The long-term behavioural consequences of prenatal stress. Neurosci. Biobehav. Rev..

[17] Mineur YS, Belzung C, Crusio WE (2006). Effects of unpredictable chronic mild stress on anxiety and depression-like behavior in mice. Behav. Brain Res.

[18] Mineur YS, Prasol DJ, Belzung C, Crusio WE (2003). Agonistic behavior and unpredictable chronic mild stress in mice. Behav. Genet..

[19] Willner P (2005). Chronic mild stress (CMS) revisited: Consistency and behavioural-neurobiological concordance in the effects of CMS. Neuropsychobiology.

[20] Campos AC, Fogaca MV, Aguiar DC, Guimaraes FS (2013). Animal models of anxiety disorders and stress. Rev. Bras. Psiquiatr..

[21] Imbe H, Iwai-Liao Y, Senba E (2006). Stress-induced hyperalgesia: Animal models and putative mechanisms. Front. Biosci..

[22] Enayati M (2012). Maternal infection during late pregnancy increases anxiety- and depression-like behaviors with increasing age in male offspring. Brain Res. Bull.

[23] Misdrahi D, Pardon MC, Perez-Diaz F, Hanoun N, Cohen-Salmon C (2005). Prepartum chronic ultramild stress increases corticosterone and estradiol levels in gestating mice: Implications for postpartum depressive disorders. Psychiatry Res..

[24] Sickmann HM, Arentzen TS, Dyrby TB, Plath N, Kristensen MP (2015). Prenatal stress produces sex-specific changes in depression-like behavior in rats: Implications for increased vulnerability in females. J. Dev. Orig. Health Dis..

[25] Weinstock M (2007). Gender differences in the effects of prenatal stress on brain development and behaviour. Neurochem. Res..

[26] Nolin SL (1996). Familial transmission of the FMR1 CGG repeat. Am. J. Hum. Genet..

[27] Vafaeie F, Alerasool M, Kaseb Mojaver N, Mojarrad M (2021). Fragile X syndrome in a female with homozygous full-mutation alleles of the FMR1 gene. Cureus.

[28] Wheeler A (2014). Anxiety, attention problems, hyperactivity, and the Aberrant Behavior Checklist in fragile X syndrome. Am. J. Med. Genet. A.

[29] Loesch DZ, Hay DA (1988). Clinical features and reproductive patterns in fragile X female heterozygotes. J. Med. Genet..

[30] Loesch DZ (2003). Effect of the fragile X status categories and the fragile X mental retardation protein levels on executive functioning in males and females with fragile X. Neuropsychology.

[31] Mazzocco MM, Kates WR, Baumgardner TL, Freund LS, Reiss AL (1997). Autistic behaviors among girls with fragile X syndrome. J. Autism Dev. Disord..

[32] Gaudissard J (2017). Behavioral abnormalities in the Fmr1-KO2 mouse model of fragile X syndrome: The relevance of early life phases. Autism Res..

[33] Gauducheau M (2017). Age-specific autistic-like behaviors in heterozygous Fmr1-KO female mice. Autism Res..

[34] Spear LP (2000). The adolescent brain and age-related behavioral manifestations. Neurosci. Biobehav. Rev..

[35] Terranova ML, Laviola G, Alleva E (1993). Ontogeny of amicable social behavior in the mouse: Gender differences and ongoing isolation outcomes. Dev. Psychobiol..

[36] Negroni J (2004). Chronic ultra-mild stress improves locomotor performance of B6D2F1 mice in a motor risk situation. Behav. Brain Res..

[37] Pardon M, Perez-Diaz F, Joubert C, Cohen-Salmon C (2000). Age-dependent effects of a chronic ultramild stress procedure on open-field behaviour in B6D2F1 female mice. Physiol. Behav..

[38] Pardon MC, Perez-Diaz F, Joubert C, Cohen-Salmon C (2000). Influence of a chronic ultramild stress procedure on decision-making in mice. J. Psychiatry Neurosci..

[39] Burkholder T, Foltz C, Karlsson E, Linton CG, Smith JM (2012). Health evaluation of experimental laboratory mice. Curr. Protoc. Mouse Biol..

[40] Moles A, D’Amato FR (2000). Ultrasonic vocalization by female mice in the presence of a conspecific carrying food cues. Anim. Behav..

[41] Moles A, Costantini F, Garbugino L, Zanettini C, D'Amato FR (2007). Ultrasonic vocalizations emitted during dyadic interactions in female mice: A possible index of sociability?. Behav. Brain Res..

[42] Wang H, Liang S, Burgdorf J, Wess J, Yeomans J (2008). Ultrasonic vocalizations induced by sex and amphetamine in M2, M4, M5 muscarinic and D2 dopamine receptor knockout mice. PLoS ONE.

[43] Warburton VL, Sales GD, Milligan SR (1989). The emission and elicitation of mouse ultrasonic vocalizations: The effects of age, sex and gonadal status. Physiol. Behav..

[44] Whitney G, Coble JR, Stockton MD, Tilson EF (1973). Ultrasonic emissions: Do they facilitate courtship of mice. J. Comp. Physiol. Psychol..

[45] Castellucci GA, Calbick D, McCormick D (2018). The temporal organization of mouse ultrasonic vocalizations. PLoS ONE.

[46] Caruso A, Ricceri L, Scattoni ML (2020). Ultrasonic vocalizations as a fundamental tool for early and adult behavioral phenotyping of Autism Spectrum Disorder rodent models. Neurosci. Biobehav. Rev..

[47] Lahvis GP, Alleva E, Scattoni ML (2011). Translating mouse vocalizations: Prosody and frequency modulation. Genes Brain Behav..

[48] Maggio JC, Whitney G (1985). Ultrasonic vocalizing by adult female mice (*Mus musculus*). J. Comp. Psychol..

[49] D'Amato FR, Moles A (2001). Ultrasonic vocalizations as an index of social memory in female mice. Behav. Neurosci..

[50] Panksepp JB (2007). Affiliative behavior, ultrasonic communication and social reward are influenced by genetic variation in adolescent mice. PLoS ONE.

[51] Wohr M (2014). Ultrasonic vocalizations in Shank mouse models for autism spectrum disorders: Detailed spectrographic analyses and developmental profiles. Neurosci. Biobehav. Rev..

[52] Mosienko V, Beis D, Alenina N, Wohr M (2015). Reduced isolation-induced pup ultrasonic communication in mouse pups lacking brain serotonin. Mol. Autism.

[53] Caligioni CS (2009). Assessing reproductive status/stages in mice. Curr. Protoc. Neurosci..

[54] Vandesquille M (2013). Working memory deficits and related disinhibition of the cAMP/PKA/CREB are alleviated by prefrontal alpha4beta2*-nAChRs stimulation in aged mice. Neurobiol. Aging.

[55] Scherr JF, Hahn LJ, Hooper SR, Hatton D, Roberts JE (2016). HPA axis function predicts development of working memory in boys with FXS. Brain Cogn..

[56] Qin M, Xia Z, Huang T, Smith CB (2011). Effects of chronic immobilization stress on anxiety-like behavior and basolateral amygdala morphology in Fmr1 knockout mice. Neuroscience.

[57] Lemaire-Mayo V, Subashi E, Henkous N, Beracochea D, Pietropaolo S (2017). Behavioral effects of chronic stress in the Fmr1 mouse model for fragile X syndrome. Behav. Brain Res..

[58] Advani T, Koek W, Hensler JG (2009). Gender differences in the enhanced vulnerability of BDNF+/− mice to mild stress. Int. J. Neuropsychopharmacol..

[59] Hodes GE (2015). Sex differences in nucleus accumbens transcriptome profiles associated with susceptibility versus resilience to subchronic variable stress. J. Neurosci..

[60] Meng F (2020). Brain-derived neurotrophic factor in 5-HT neurons regulates susceptibility to depression-related behaviors induced by subchronic unpredictable stress. J. Psychiatr. Res..

[61] Mueller BR, Bale TL (2007). Early prenatal stress impact on coping strategies and learning performance is sex dependent. Physiol. Behav..

[62] Schwendener S, Meyer U, Feldon J (2009). Deficient maternal care resulting from immunological stress during pregnancy is associated with a sex-dependent enhancement of conditioned fear in the offspring. J. Neurodev. Disord..

[63] Takayanagi Y, Onaka T (2021). Roles of oxytocin in stress responses, allostasis and resilience. Int. J. Mol. Sci..

[64] Marchisella F (2021). Exposure to prenatal stress is associated with an excitatory/inhibitory imbalance in rat prefrontal cortex and amygdala and an increased risk for emotional dysregulation. Front. Cell Dev. Biol..

[65] Nakai N, Overton ETN, Takumi T (2021). Optogenetic approaches to understand the neural circuit mechanism of social deficits seen in autism spectrum disorders. Adv. Exp. Med. Biol..

[66] Gangopadhyay P, Chawla M, Dal Monte O, Chang SWC (2021). Prefrontal-amygdala circuits in social decision-making. Nat. Neurosci..

[67] Branchi I (2009). The mouse communal nest: Investigating the epigenetic influences of the early social environment on brain and behavior development. Neurosci. Biobehav. Rev..

[68] Moles A, Rizzi R, D'Amato FR (2004). Postnatal stress in mice: Does "stressing" the mother have the same effect as "stressing" the pups?. Dev. Psychobiol..

